# KNOW-KT (KoreaN cohort study for outcome in patients with kidney transplantation: a 9-year longitudinal cohort study): study rationale and methodology

**DOI:** 10.1186/1471-2369-15-77

**Published:** 2014-05-09

**Authors:** Jaeseok Yang, Joongyup Lee, Kyu Ha Huh, Jae Berm Park, Jang-Hee Cho, Sik Lee, Han Ro, Seung-Yeup Han, Young Hoon Kim, Jong Cheol Jeong, Byung-Joo Park, Duck Jong Han, Sung-Bae Park, Wookyung Chung, Sung Kwang Park, Chan-Duck Kim, Sung Joo Kim, Yu Seun Kim, Curie Ahn

**Affiliations:** 1 Transplantation Center, Seoul National University Hospital, Seoul, Republic of Korea; 2 Medical Research Collaborating Center, Seoul National University Hospital, Seoul, Republic of Korea; 3 Department of Transplantation Surgery, Severance Hospital, Yonsei University Health System, Seoul, Republic of Korea; 4 Department of Surgery, Sungkyunkwan University, Seoul Samsung Medical Center, Seoul, Republic of Korea; 5 Department of Internal Medicine, Kyungpook National University Hospital, Seoul, Republic of Korea; 6 Department of Internal Medicine, Chonbuk National University Hospital, Seoul, Republic of Korea; 7 Department of Internal Medicine, Gachon University, Gil Hospital, Seoul, Republic of Korea; 8 Department of Internal Medicine, Keimyung University, Dongsan Medical Center, Seoul, Republic of Korea; 9 Department of Surgery, Ulsan University, Seoul Asan Medical Center, Seoul, Republic of Korea; 10 Department of Preventive Medicine, Seoul National University College of Medicine, Seoul, Republic of Korea; 11 Department of Internal Medicine, Seoul National University College of Medicine, Seoul, Republic of Korea

**Keywords:** Cohort study, Complication, Kidney transplantation, KNOW-KT, Risk factor

## Abstract

**Background:**

Asian patients undergoing kidney transplantation (KT) generally have better renal allograft survival and a lower burden of cardiovascular disease than those of other racial groups. The KNOW-KT aims to explore allograft survival rate, cardiovascular events, and metabolic profiles and to elucidate the risk factors in Korean KT patients.

**Methods:**

KNOW-KT is a multicenter, observational cohort study encompassing 8 transplant centers in the Republic of Korea. KNOW-KT will enroll 1,000 KT recipients between 2012 and 2015 and follow them up to 9 years. At the time of KT and at pre-specified intervals, clinical information, laboratory test results, and functional and imaging studies on cardiovascular disease and metabolic complications will be recorded. Comorbid status will be assessed by the age-adjusted Charlson co-morbidity index. Medication adherence and information on quality of life (QoL) will be monitored periodically. The QoL will be assessed by the Kidney Disease Quality of Life Short Form. Donors will include both living donors and deceased donors whose status will be assessed by the Kidney Donor Risk Index. Primary endpoints include graft loss and patient mortality. Secondary endpoints include renal functional deterioration (a decrease in eGFR to <30 mL/min/1.73 m^2^), acute rejection, cardiovascular event, albuminuria, new-onset diabetes after transplant, and QoL. Data on other adverse outcomes including episodes of infection, malignancy, recurrence of original renal disease, fracture, and hospitalization will also be collected. A bio-bank has been established for the acquisition of DNA, RNA, and protein from serum and urine samples of recipients at regular intervals. Bio-samples from donors will also be collected at the time of KT. KNOW-KT was registered in an international clinical trial registry (NCT02042963 at http://www.clinicaltrials.gov) on January 20^th^, 2014.

**Conclusion:**

The KNOW-KT, the first large-scale cohort study in Asian KT patients, is expected to represent the Asian KT population and provide information on their natural course, complications, and risk factors for complications.

## Background

The number of end stage renal disease (ESRD) patients has been increasing worldwide. In ESRD patients, the benefits in relation to survival and quality of life are greater with kidney transplantation (KT) than with dialysis. As the acute rejection rate is currently decreasing, long-term complications have become an important issue in KT patients. However, the incidence and patterns of complications in Asian KT patients may be different from those in Western countries. For example, a prospective cohort study for Chinese general population [1] and a retrospective study for Korean transplant patients [2] reported a lower incidence of ischemic heart disease than that reported in Western countries. Therefore, it is necessary to analyze outcomes and complications in KT patients in Asian countries.

The number of KTs has been increasing in Korea, and the incidence and prevalence are 33.5 and 272.3 per million people, respectively [3]. Outcomes of KT in Korea are very good in both graft and patient survival rates. Furthermore, deceased donor (DD) KT program is the most active in Korea among Asian countries with the establishment of donor action programs, and the proportion of DDKTs reached approximately 43.1% in 2012. In this sense, a systemic analysis of Korean transplant data could be valuable as representative data of the Asian population.

After transplantation, renal function tends to decrease with time. More than 60% of KT patients have an estimated glomerular filtration rate (eGFR) less than 60 mL/min/1.73 m^2^ at 1 year after transplantation, and a mild degree of proteinuria or hematuria is commonly seen in KT patients [4]. Therefore, more than 90% of KT patients are indeed chronic kidney disease patients [5]. KT patients have various complications that are derived from chronic immunosuppression or chronic kidney disease status. Cardiovascular events, malignancy, infection, and chronic rejection are the main cause of mortality or allograft failure in KT patients, and the patients also suffer from metabolic and psychosocial complications and mineral bone disorder. Analysis of the incidence and risk factors for these complications is necessary to control complications and improve transplant outcomes.

Several prospective, observational cohort studies have been performed in the Europe and North America [6-9]; however, well-designed studies in other regions are lacking. The KoreaN cohort study for Outcome in patients With KT: A 9-year Longitudinal cohort study of the Korean adult KT patients (KNOW-KT), funded by Korea Center for Disease Control and Prevention (KCDC), was launched in 2012 by a group of nephrologists, surgeons, epidemiologists, and biostatisticians in Korea. We aimed to establish an adult KT cohort; to investigate the renal allograft outcomes, mortality, and complications; to explore traditional or nontraditional risk factors for morbidity and mortality; and to establish bio-bank to integrate clinical and biological information. Here, we report the design and methodology of the KNOW-KT.

## Methods

### Organization

The KNOW-KT is being conducted in eight university-affiliated Korean transplant centers (four in Seoul City, one in Incheon city, one in Chonbuk Province, and two in Kyoungbuk Province), and involves a team of epidemiologic experts and a bio-bank in the KCDC. Epidemiologists and biostatisticians in the Medical Research Collaborating Center of Seoul National University Hospital (MRCC) are responsible for data management and statistical analysis. The KNOW-KT runs the steering committee to guide the study. The study is supervised by the Chronic Kidney Disease Advisory Committee composed by the members of the KCDC and the Korean Society of Nephrology.

### Study objectives

The primary objective of the present study is to delineate the predictors of graft failure, patient mortality, and cardiovascular and metabolic complications after KT. The secondary objective is to describe various aspects of other complications based on prospectively collected data.

### Study design and study population

This is a prospective, observational cohort study. Total enrollment population will be 1,000 adult KT recipients and their corresponding donors. Study recruitment will be performed over 4 years from 2012 to 2015. The study population will be followed up for 9 years or until their death, graft failure, or drop out. Study inclusion and exclusion criteria are shown in Table 1. Participating subjects will visit each center according to the follow-up schedule. At each visit, the subjects will undergo scheduled tests and be reviewed by the investigators for recent medical history and event occurrence. Efforts will be made in order to prevent drop-outs, i.e., providing free medical tests, frequent telephone calls and dietary education.

**Table 1:** Inclusion and exclusion criteria of the KNOW-KT

**Inclusion criteria**	**Exclusion criteria**
Kidney transplant recipients and donors	Simultaneous multiple organ transplantation
Age > 18, both donors and recipients	En-bloc KT recipients
Agreed to participate and sign the consent form	Comorbidities of liver cirrhosis or interstitial lung disease at the time of transplant

### Collection of data

Baseline data will be collected during the pre-transplant screening period, which include socio-demographic information (age, sex, smoking history, history of alcohol drinking), information on quality of life (QoL), socio-economic status, educational level, physical activity, health behaviors, and health care facility utilization. Comorbid status will be assessed by the age-adjusted Charlson co-morbidity index [10]. The QoL will be assessed by the Kidney Disease Quality of Life Short Form (KDQoL-SF) [11]. The KDQoL-SF consists of generic core (physical component summary and mental component summary) and kidney disease-targeted domains (symptoms/problems, burden of kidney disease, and effects of kidney disease). Generic core represents the physical and mental aspects of QoL, and kidney disease-targeted domains focus on disease-related concerns. At the time of KT, the following clinical parameters will be collected; (1) general information about transplant (date of transplantation, number of transplant experience, donor-recipient relationship and desensitization) (2) recipient information (date of birth, sex, cause of ESRD, comorbidities such as diabetes mellitus and hypertension, history of malignancy, history of cardiovascular diseases, and medications including immunosuppressant) (3) donor information (date of birth, sex, body weight, height, comorbidities such as hypertension, history of malignancy). Donors include both living donors and DDs. DDs include brain death donors of standard and expanded criteria, and donation after cardiac death. The quality of DD kidneys will be assessed by the Kidney Donor Risk Index [12]. Physical examination including anthropometric measurements (height, weight, waist-to-hip ratios) and measurements of the resting office blood pressure and pulse pressure will be conducted. Laboratory tests include serum levels of calcium, phosphorus, parathyroid hormone, total cholesterol, high-density lipoprotein cholesterol, triglycerides, low-density lipoprotein cholesterol (calculated), hemoglobin, glycated hemoglobin, and C-reactive protein. Immunologic evaluation includes human leukocyte antigen (HLA) typing, performing HLA crossmatch (complement-dependent cytotoxicity-based method and flow cytometry-based method), and measuring panel reactive antibody levels. Pre-transplant echocardiography, measurement of pulse-wave velocity and ankle brachial index, and coronary calcium computed tomography will be performed as cardiac evaluations.

Complication events will be collected annually as an event-based report including the date in which complication occurred. Follow-up examinations are scheduled once a year. Evaluation schedule of the KNOW-KT at each visit (Table 2) summarized details regarding the schedules for study visits, tests, and questionnaires. Immunosuppressive medications at discharge will be recorded as a baseline, and then they will be recorded annually. Medication adherence will be monitored annually using Health Questionnaires.

**Table 2:** Evaluation schedule of the KNOW-KT at each visit

	Visit*										
Parameter	Screening	Baseline	1	2	3	4	5	6	7	8	9
Informed consent	O										
Medical History	O										
Eligibility confirmation	O										
Recent events			O	O	O	O	O	O	O	O	O
Medications		O	O	O	O	O	O	O	O	O	O
Health Questionnaires		O	O	O	O	O	O	O	O	O	O
KDQOL questionnaires		O		O		O		O		O	
Blood pressure		O	O	O	O	O	O	O	O	O	O
Anthropometry		O	O	O	O	O	O	O	O	O	O
CBC, chemistry		O	O	O	O	O	O	O	O	O	O
IDMS-serum Cr, eGFR		O	O	O	O	O	O	O	O	O	O
Cystatin C		O	O	O	O	O	O	O	O	O	O
HbA1c (diabetic subjects)		O	O	O	O	O	O	O	O	O	O
Lipid, CRP, iron profile		O	O	O	O	O	O	O	O	O	O
Intact PTH, vitamin D^1^		O	O	O	O	O	O	O	O	O	O
Urinalysis		O	O	O	O	O	O	O	O	O	O
Urine protein/creatinine		O	O	O	O	O	O	O	O	O	O
EKG, chest X ray		O	O	O	O	O	O	O	O	O	O
Serum BK real time PCR			O				O				
PRA I & II		O	O				O				
HLA crossmatch		O									
HLA typing		O									
Viral serology^2^		O									
LS spine Lateral X-ray for vascular calcification		O	O	O	O	O	O	O	O	O	O
Pulse wave velocity, Ankle-brachial index		O			O			O			
Echocardiography		O			O			O			
MDCT for coronary calcium score		O						O			
Bone mineral density		O			O			O			
DNA sample*		O									
Serum/urine sample		O	O		O		O				
RNA sample			O		O		O				

CBC, complete blood count; CMV, cytomegalovirus; CRP, C-reactive protein; HLA, human leukocyte antigen; IDMS-serum cr, serum creatinine measurement using IDMS-traceable method; KDQOL, Kidney Disease Quality of Life; MDCT, multidetector computed tomography; PRA, panel reactive antibody; PTH, parathyroid hormone. ^1^25-hydroxy- and 1,25-dihyroxy-vitamin D. ^2^HBsAg, anti-HBs, anti-HCV, and anti-CMV IgG.

All the reported data will be collected in a predefined case report format and subsequently entered into a web-based electronic data-warehouse. The electronic data input and management system for the KNOW-KT was developed by the data management division in the MRCC.

### Study outcomes

Primary endpoints include graft loss and patient mortality. Graft loss is defined as requiring maintenance dialysis for more than 3 months or re-transplantation. The cause of graft loss will be pursued. Patient mortality will be classified into cardiac, noncardiac, and unknown death. Subjects who drop out from the study will be traced for this information with the help of National Health Insurance Cooperation and Korea Statistical Information Service.

Secondary endpoints include renal functional deterioration, acute rejection, cardiovascular event, albuminuria, new-onset diabetes after transplant, and QoL. Renal functional deterioration is defined as a decrease in eGFR to less than 30 mL/min/1.73 m^2^ after transplantation. Serum creatinine will be measured by an isotope-dilution mass spectrometry (IDMS)-traceable method. Estimations for defining renal deterioration will be based on both the four-variable Modification of Diet in Renal Disease formula [13], and Chronic Kidney Disease Epidemiology Collaboration creatinine equation [14]. Cardiovascular events include myocardial infarction, coronary revascularization, stroke and new onset or aggravation of congestive heart failure.

Data on other adverse outcomes including episodes of infection, malignancy, recurrence of original renal disease, fracture, chronic liver disease, and hospitalization will also be collected.

### Reference populations for the evaluation of chronic complications

To compare the risk of developing chronic medical complications, we plan to use two reference populations. One consists of approximately 3,000 CKD patients who are enrolled in the KoreaN cohort study for Outcome in patients With Chronic Kidney Disease (KNOW-CKD), and the other is the general Korean population compiled from the compulsory national health insurance program without any claims under the diagnosis of CKD. Data from the KNOW-CKD will be used for a pooled analysis to provide the incidence rate ratios adjusting for various risk factors. For the comparison between the general population and the KNOW-KT, standardized incidence rates will be presented.

### Biospecimen banking

As baseline DNA samples, 4 mL of the whole blood from recipients, collected in ethylenediaminetetraacetic acid tube will be used. Another 10 mL of whole blood will be collected in serum separation tube and centrifuged within 1 hour for serum sample stock. Ten mL of morning first-voided urine samples will be taken for urine sample stock and urine sediments will also be prepared by centrifugation. Aliquots of the serum and urine samples will be stored in a deep freezer (−70 °C). Serum and urine samples will be collected at baseline and year 1, 3, and 5 after enrollment. For RNA extraction and storing, 10 mL of whole blood will be collected in PAXgene® tubes (Qiagen Inc., Valencia, CA, USA) at year 1, 3, and 5 visits. DNA samples from donors will be collected in the same manner as in the recipients. Sampling, delivery, and storage of samples will be under strict monitoring throughout the study period.

### Ethical considerations

The study is conducted under the Declaration of Helsinki. The regional ethical committee and Institutional Review Board of each participating center approved the study protocol. Informed consent in written form is essential to recruit study population, for both donor and recipients. It was also registered in an international clinical trial registry (NCT02042963 at http://www.clinicaltrials.gov) on January 20^th^, 2014.

### Statistical considerations

Descriptive analysis will be performed for baseline data. Normally distributed continuous variables will be presented as mean ± standard deviation. For nonparametric continuous variables, median with interquartile ranges will be used as the summarizing values. Categorical data will be presented with numbers and proportions. Comparison of continuous variables will be conducted with t-test or Wilcoxon rank-sum test, and categorical variables will be compared using chi-squared test or Fisher’s exact test as appropriate.

Survival time of grafts or patients, time to cardiovascular outcomes (myocardial infarction, ischemic stroke, etc.) and renal outcomes (graft loss, renal functional deterioration, acute rejection, etc.) will be estimated using Kaplan-Meier curves. Cox proportional hazard models will be used for comparison for the incidence of those outcomes among groups with and without each risk factor. However, patient death is a competing cause of renal outcomes or cardiovascular outcomes. Therefore, we will adopt competing risk models in evaluating the contributions of risk factors for those outcomes. For time-varying covariates that break the proportional hazard assumption, an extended Cox model to compare the risk of outcomes will be used.

Since more than 60% of KT patients have CKD at 1 year after transplantation [4], and CKD patients often have a non-linear eGFR decline or a prolonged period of non-progression [15], the pattern of renal functional deterioration will be explored using the Bayesian smoothing technique [16]. We will compare eGFR decline in serial measurements using a mixed linear model considering the longitudinal nature of our data. The generalized estimating equation will be used for estimating the slope of the eGFR in the area of steady and linear eGFR decline.

We will enroll 1,000 KT patients from participating centers. We assumed that the incidence of cardiovascular complications at 5 years would be 22% [17]. With that assumption, the sample size will detect a relative risk of 1.46 or more by a risk factor with a prevalence of 50% at 5% alpha and 20% beta in an analysis using Cox regression model. Assuming the same significance level and statistical power, for a risk factor with prevalence of 12%, the sample size would detect a relative risk of 2.00 or higher as significant from same statistical model. Analysis will be performed by using SAS 9.3 (SAS Institute, Cary, NC, USA). Statistical analysis will be 2-sided, and p-value < 0.05 will be considered statistically significant.

### Study status

The KNOW-KT has enrolled 535 patients until now, and plans to enroll a total of 1,000 patients by 2015.

## Discussion

Although modern immunologic assessments and potent immunosuppression markedly decreased the acute rejection rate in KT, it fails to suppress chronic rejection due to immunologic and non-immunologic factors; this has becomes a major obstacle for long-term graft survival. Furthermore, as both graft and patient survival improve, KT patients, who are often in the early stages of CKD, become exposed to chronic immunosuppression. Therefore, KT patients can develop many medical complications such as cardiovascular events, metabolic complications, infection, malignancy, and mineral bone disorders as well as chronic rejection. Indeed, chronic medical complications have become a major cause of morbidity and mortality in KT patients.

Antibody-mediated injury is an important cause of immunologic injury in chronic rejection or chronic allograft nephropathy. Because noncompliance is one of main causes of antibody formation, compliance monitoring together with antibody monitoring is included in our prospective study. Hypertension, hyperlipidemia, and diabetes, so called non-immunologic risk factors for chronic allograft nephropathy, are common after KT and are reported to induce chronic damage to allografts.

The nonimmunologic risk factors for chronic allograft nephropathy are among traditional risk factors for cardiovascular disease. Cardiovascular events have been reported to be the most common cause of death in KT patients in Western countries. However, many studies show that racial/ethnic disparities exist in mortality and cardiovascular outcome among CKD patients [18-21]. Transplant outcomes seem to differ according to racial differences [22,23]. Asian KT patients generally have better renal allograft survival and a lower burden of cardiovascular disease than other racial groups. The KNOW-KT, the first large-scale cohort study in Asian KT patients, aims to explore allograft survival rate, cardiovascular events, and metabolic profiles and to investigate the risk factors in Asian or Korean KT patients.

Risk for malignancy is higher in CKD patients than in the general population [24]. Moreover, the risk for malignancy is higher in KT patients than in CKD patients due to chronic immunosuppression. The relative prevalence of common malignancy differs according to region. For example, gastric and hepatic cancers are the most common types of cancer in Korea, whereas they are not as common in Western countries. Therefore, malignancy patterns in KT patients could differ between Asian and Western countries. Infectious complications are also one of the main complications after transplantation. There may be differences in immunosuppressive protocols or prophylaxis policy due to differences in health insurance coverage or disease prevalence. These potential differences could result in different patterns of infection in Korean KT patients. Analysis of local infection profiles can help in redirecting prophylactic policy for KT patients in a specific country.

The KNOW-KT will evaluate the complication profiles of mineral-bone disorders across various renal functions. The KNOW-KT will also evaluate the socio-economic burden and impact on the quality of life. Comparison of the KNOW-KT with other large-scale KT cohorts through international collaboration may provide a platform for future research.

Various biomarkers from the serum and urine samples will be explored for the risk prediction of adverse consequences. Genetic and epigenetic factors will be sought. Interaction between donor factors and recipient factors is a unique feature in transplant cohorts and cannot be seen in conventional CKD cohorts. Especially, analysis of bio-samples from both living donors and DDs together with recipient bio-samples could be one of the strengths of the present cohort study.

The Collaborative Transplant Study (CTS) based in Europe [25], the Scientific Registry of Transplant Recipients (SRTR) in USA [26], and the Australia and New Zealand Dialysis and Transplant (ANZDATA) registry are large-scale registries [27]. All these large cohorts have strengths related to the large sample size and the fact that they are representative as nationwide database, whereas they also have limitations inherent to as registry data, such as simplicity of the outcome data and the heterogeneity of clinical follow-up data. They usually do not collect bio-samples that are matched with clinical data. However, the KNOW-KT clearly defined outcomes and carefully monitors study processes in order to meet predefined protocols and establish a bio-bank together with clinical data.

There are a few limitations in the KNOW-KT. First, the KNOW-KT will enroll a relatively small number of patients compared with the larger-scale registry database or the cohort studies in Western countries. Therefore, we should expand out cohort later in order to confirm some of the findings. Second, all routine laboratory and sampling procedures are processed in individual local transplant centers, although transport and storage of biospecimens are managed by a central laboratory. However, standards, laboratory methods, measurement units, and detection limits are generally consistent among these local laboratories; therefore, the discrepancies among procedures at individual laboratories will only have limited effects on the quality of collected data, if any. Third, we do not mandatorily collect kidney biopsy samples; instead, it depends on the policy of individual center. Nevertheless, the KNOW-KT is the first large-scale KT cohort study in Asian KT patients. Future larger-scale studies could confirm the major findings of this study.

As the first large-scale Asian KT cohort study to be performed longitudinally for up to 9 years, the KNOW-KT will help clarify the natural course, complication profiles, and the risk factors of the Asian KT population. Analyzing this cohort and comparing with other cohort studies in Western countries will provide information about the impact of race on transplant outcomes and a comprehensive insight into KT.

## Competing interest

The authors declare that they have no competing interest.

## Authors’ contributions

JY, JL, JCJ, and CA participated in the design and coordination of the study. JL and Kyoungha Seo in the KNOW-KT Study Group are in charge of biostatistical analysis. KHH, JBP, JHC, SL, HR, SYH, YHK, and BJP reviewed the study protocol. JY, KHH, JBP, JHC, SL, HR, SYH, YHK, BJP, DJH, JCJ, SBP, WC, SKP, CDK, SJK, YSK, and CA were involved in patient enrollment, and data collection. JY, JL, and JCJ drafted the manuscript. All of the authors read, revised, and approved the final manuscript.

## Authors’ information

The KNOW-KT Study Group includes the following researchers:

Clinical centers

*Seoul National University, Seoul National University Hospital*

Curie Ahn, M.D. (PI)

Jaeseok Yang, M.D.

Jong Cheol Jeong, M.D.

*Yonsei University Health System, Severance Hospital*

Yu Seun Kim, M.D.

Myung Soo Kim, M.D.

Kyu Ha Huh, M.D.

*Sungkyunkwan University, Seoul Samsung Medical Center*

Sung Joo Kim, M.D.

Jae Berm Park, M.D.

*Kyungpook National University Hospital*

Chan-Duck Kim, M.D.

Jang-Hee Cho, M.D.

*Chonbuk National University Hospital*

Sung Kwang Park, M.D.

Sik Lee, M.D.

*Gachon University, Gil Hospital*

Wookyung Chung M.D.

Han Ro, M.D.

*Keimyung University, Dongsan Medical Center*

Sung-Bae Park, M.D.

Seung-Yeup Han, M.D.

Eunah Hwang, M.D.

Wooyeong Park, M.D.

*Ulsan University, Seoul Asan Medical Center*

Duck Jong Han, M.D.

Young Hoon Kim, M.D.

Epidemiology and Biostatistics

Department of Preventive Medicine, Seoul National University College of Medicine

Byung-Joo Park, M.D.

Medical Research Collaborating Center, Seoul National University Hospital and Seoul National University College of Medicine

Joongyup Lee, M.D.

Kyoungha Seo, M.S.

Coordinating Center

Medical Research Collaborating Center, Seoul National University Hospital and Seoul National University College of Medicine

Joongyup Lee, M.D.

Heejung Ahn, R.N.

Seong-eun Kim, R.N.

Central Laboratory

Donghee Seo, M.D., Lab Genomics, Korea

Dae Yeon Cho, Ph.D., Lab Genomics, Korea

Biobank

Korea Biobank, Korea Centers for Disease Control and Prevention, Osong, Korea

Korea Centers for Disease Control and Prevention

Dukhyoung Lee, M.D.

Hyekyung Park, M.D. (Project Officer)

Eunkyeong Jung, M.D.

Suyeon Jeong

Eunmi Ahn

Sil-Hae Sung

## Pre-publication history

The pre-publication history for this paper can be accessed here:

http://www.biomedcentral.com/1471-2369/15/77/prepub
